# Estimating mouse and rat use in American laboratories by extrapolation from Animal Welfare Act-regulated species

**DOI:** 10.1038/s41598-020-79961-0

**Published:** 2021-01-12

**Authors:** Larry Carbone

**Affiliations:** University of California San Francisco, San Francisco, CA 94117 USA

**Keywords:** Biological models, Experimental models of disease, Preclinical research, Animal biotechnology

## Abstract

Alone among Western nations, the United States has a two-tier system for welfare protections for vertebrate animals in research. Because its Animal Welfare Act (AWA) excludes laboratory rats and mice (RM), government veterinarians do not inspect RM laboratories and RM numbers are only partially reported to government agencies^1^. Without transparent statistics, it is impossible to track efforts to reduce or replace these sentient animals’ use or to project government resources needed if AWA coverage were expanded to include them. I obtained annual RM usage data from 16 large American institutions and compared RM numbers to institutions’ legally-required reports of their AWA-covered mammals. RM comprised approximately 99.3% of mammals at these representative institutions. Extrapolating from 780,070 AWA-covered mammals in 2017–18, I estimate that 111.5 million rats and mice were used per year in this period. If the same proportion of RM undergo painful procedures as are publicly reported for AWA-covered animals, then some 44.5 million mice and rats underwent potentially painful experiments. These data inform the questions of whether the AWA needs an update to cover RM, or whether the NIH should increase transparency of funded animal research. These figures can benchmark progress in reducing animal numbers in general and more specifically, in painful experiments. This estimate is higher than any others available, reflecting the challenges of obtaining statistics without consistent and transparent institutional reports.

## Introduction

How many animals do scientists use in American laboratories every year? Are numbers increasing, decreasing or staying the same? How successful are efforts to reduce animal numbers, especially in potentially painful or distressing protocols? No one knows. The United States collects data on a subset of species defined as “animals” in the Animal Welfare Act (AWA) but has no system for collecting statistics on scientists’ use of mice, rats or fish. Various estimates are available, but either lack evidence or are based on extrapolations from countries such as Canada, the United Kingdom, Switzerland and the European Union that have greater transparency and whose laboratory animal welfare laws do not exclude the most numerous species^1–7^.

In 1970, Congress amended the AWA to require annual reports of all research animals, in categories corresponding to the pain and distress the animals experienced. By federal policy at that time, and later, by a congressional act in 2002, the AWA excludes most rats and mice (RM) from its coverage. Congress developed a system to allow tracking of efforts to reduce animal use in general and use in painful experiments in particular^1^. Reduction of animal numbers is one of the “Three Rs” humane alternatives of laboratory animal welfare, whether via replacement of animals altogether or designing experiments to minimize pain and distress and minimize the number of animals undergoing pain and distress^8,9^.

At a national level, two federal agencies, the USDA and the NIH, and also the private non-profit accrediting organization, AAALAC-International, collect animal-use data from the institutions they oversee, but none of them covers all United States RM. AAALAC only covers facilities that voluntarily seek its accreditation and NIH only covers animals in laboratories with federal grants (such as from NIH, the National Science Foundation, and the Department of Defense). USDA administers the AWA, which excludes RM bred for research; it would cover wild-caught mice and rats but not their lab-born offspring. Thus, none of the three counts all United States RM usage. While AAALAC and NIH collect RM-use statistics for the laboratories they cover, they do not compile them into a national tally for public access and they allow for varied methods of accounting for the animals reported to them. AAALAC’s confidentiality policies do not allow release of individual or aggregate statistics NIH complies with Freedom of Information requests one-by-one for facilities it oversees, but does not compile or share aggregate statistics.

USDA’s AWA enforcement includes unannounced inspections by government veterinarians, on-site investigation of whistleblower complaints, and transparent publication of animal use and of inspection reports. Bringing RM into the AWA under USDA coverage would give these animals this level of oversight that primates, dogs, hamsters and other mammals receive. Without solid data on RM, it is impossible to argue that their numbers are too large to ignore, or to project the level of funding and staffing USDA would require to oversee their welfare in laboratories.

To estimate RM use in the US, I sought to extrapolate from the USDA’s published statistics on AWA-covered mammals used in research and reported in columns C, D and E of facilities’ annual reports. I received the annual RM usage, as reported in their AAALAC annual reports, from 16 of the 30 top-funded NIH public and private research grant recipient institutions, 11 responding to public records requests and 5 voluntarily sharing their data. The 16 institutions together received about 22% of NIH research funding for the reporting year. Comparing AAALAC RM data from 16 institutions with their publicly accessible USDA annual reports, I established that at these publicly funded large institutions, RM comprised 99.3% (range 97.3–99.9%; median = 99.4%) of the mammals used annually. Applying this percentage to USDA’s 2017–2018 compiled nationwide total of 780,070 “animals” I derived an estimate of approximately 111.5 million RM (Table 1).

**Table 1:** Animal numbers from 16 institutions’ annual reports.

Institution	RM annual use	AWA “animals” 2017–18	RM %	RM average daily inventory	RM annual use-to-daily inventory
1	1,050,391	8855	99.2		
2	592,037	1819	99.7	52,849	11.7
3	480,457	2222	99.5		
4	296,947	1596	97.3	199,552	1.5
5	192,220	1762	99.1	56,787	3.4
6	568,718	1308	99.8		
7	336,234	736	99.8	116,211	2.9
8	250,172	1142	99.5	96,170	2.6
9	305,171	4014	98.7	65,278	4.67
10	77,300	112	99.9		
11	389,107	466	99.9		
12	91,722	1714	98.1	61,945	1.5
13	133,243	566	99.6		
14	306,078	5698	98.1	14,079	21.7
15	254,731	6407	97.5	254,847	1
16	236,296	607	99.7	111,404	2.1
Total	5,560,824	39,024	99.3	1,029,122	5.4

“RM annual use” the annual reports to AAALAC-International, which institutions shared with the author. “AWA ‘animals’” are publicly available from the United States Department of Agriculture. “RM %” is the percentage of an institutions AWA animals plus annual RM use that is RM. “Rat-Mouse average daily inventory” obtained via Freedom of Information request to the National Institutes of Health Office of Laboratory Animal Welfare; the numbers on those reports do not specify whether it is the number of cages, the counted number of animals, or an estimate of the number of animals based on the standing inventory of cages.

I confirmed with AAALAC administration that they do not release aggregate statistics on numbers of animals reported to them in facilities’ annual reports, nor do they release individual institutions’ reports. I likewise confirmed with the NIH OLAW that they do not compile and report facilities’ average daily animal censuses, as submitted every 4 years in facilities’ applications to renew their Assurance of Animal Welfare. Individual institutions’ NIH materials are available via Freedom of Information request to the NIH. Institutions can release their own AAALAC annual reports, and public universities may be required to release their AAALAC annual reports under their state’s public records laws.

I contacted 12 public universities on the list of NIH’s top 30 grant recipients, requesting their reported use of RM from their most recent annual report to AAALALC, citing their state’s open records requirements; 11 complied and provided their data. Additionally, I wrote to the animal care and use office or the Attending Veterinarian of the other 18 top-funded institutions in NIH’s top 30, including 1 public institution that is exempt under its state rules, with the same request. I reminded my correspondents that their use of monkeys, dogs and other USDA-covered species was already publicly available and so I would only need mouse and rat numbers.

I summed the numbers of USDA-reported “animals,” mice, and rats. The 16 responding institutions together reported using 39,024 AWA-covered “animals,” which is 5% of the 780,070 national total for 2017–2018. Total RM use in the annual reports I received was 556,0824, allowing calculation of RM use as 99.3% of mammals at the responding institutions, which is the basis for extrapolating to total national RM use, and also for the numbers of RM in the various USDA/AWA “pain categories”.

Among the 14 that gave separate counts, mice averaged 97.3% of the rat-mouse total (range 95.7 to 99.6%). Two responding institutions did not separate mice from rats, so all analysis in this paper combines the two species.

While total numbers used may be worth knowng, it is at least equally important to know how many of those animals suffer pain or distress in their use. The AWA requires that “animals” be reported in USDA Pain Categories. Category C includes animals whose experimental use causes little pain or distress and no need for pain-relieving analgesics (“No Pain No Drugs” or “NPND” in USDA’s shorthand). Categories D and E include procedures that can cause significant pain or distress; in Category D, drugs are used to treat the pain or distress (“With Pain With Drugs” or “WPWD” in USDA’s shorthand) while in Category E (“With Pain, No Drugs” or “WPND”) scientists do not use drugs to mitigate the pain or distress^10^. If RM distribute into categories in the same proportions as AWA-covered “animals,” then approximately 67 million were in Category C, with 44.5 million on studies with painful or distressing procedures (36 million in Category D, and 8 million in Category E). This reflects USDA’s reported percentages of approximately 60% C, 32% D, and 7% E (Table 2).

**Table 2:** Distribution of “used animals” in USDA “Pain Categories” C, D, and E: 2017–2018.

	AWA-covered animals reported	Rats and mice estimated
Category C “No pain no drugs”	471,037	67 million
Category D “With pain, with drugs”	253,002	36 million
Category E “With pain, with drugs”	56,031	8 million
Total C + D + E	780,070	111 million

AWA-covered animals and percentages from USDA’s summarized nationwide AWA statistics. Estimate of approximate numbers of RM used in each category if their pain categories are the same as AWA-covered animals, based on data that RM are approximately 99.3% of all mammals used in US laboratories. Category descriptors of pain and drug use are those used by the USDA.

In this project, I have derived an estimate of United States RM usage using a sampling method that future studies can replicate to evaluate trends. This required making choices among different methods and possible comparative data and making various assumptions and definitions, which I discuss below. My decisions reflect my 18 years’ experience preparing AWA, NIH and AAALAC reports for one of the 16 universities in my dataset.

One alternative method would be to compare institutions’ “average daily census” as reported every 4 years to NIH as a marker of annual RM use (Table 1). This has the advantage that these reports are available from NIH via FOIA requests, unlike the AAALAC accreditation reports I obtained. Like my approach, this is limited to federally funded institutions that report to the NIH. Goodman et al.used this approach to analyze US animal numbers at the 25 largest NIH grant recipients. Their approach likely gave them quality longitudinal trends for a 15 year period (in which they found a trend of increasing animal use with time), but does not readily translate to total animal use nationally, or even at the institutions whose records they examined, given the uncertainty in generating annual use from daily inventory^11,12^.

Similar to Goodman’s approach, I submitted Freedom of Information requests to the NIH on the 11 responding institutions covered by state public records rules, asking for the “average daily inventory” data in the 11 institutions’ Assurance of Animal Welfare documents on file with the NIH OLAW. The NIH has flexible reporting requirements for reporting “average daily census” and accepts these data as the average daily population of individually counted animals, the daily inventory of occupied cages, or extrapolations from number of cages to number of animals, with no requirement to state precisely what the institution is reporting. Nor does “average daily census” necessarily translate to “annual usage.” I obtained daily inventory statistics for 11 institutions (Table 1). One institution reported counting cages and the other ten did not specify. In the ten instances where I had both an institution’s NIH daily RM inventory data and its AAALAC annual use data, the ratio of used-to-inventory ranged broadly, from 1 to 21.7 (mean = 5.3), suggesting that average daily census is too variably reported to the NIH to currently be useful for estimating total annual usage. In my former employment, administering these reports for the University of California San Francisco, I found that one occupied cage on inventory represented a standing population of about three mice and an annual use of at least ten mice. Low ratios of use-to-inventory are suspicious; for a ratio of 1 (as one large institution reported) to be correct, the same individual animals would have to be present in the facility for the whole year, with no births, deaths, or purchases, i.e., no population turnover. Moreover, I can report that absent explicit reporting instructions from NIH or AAALAC, institutions may change their own standards of what they count and report year by year: average cage count, average daily population, total animals acquired or removed, whether or not the animals were actually used on experiments. This presents a challenge to anyone hoping to use Goodman’s data as a benchmark for trending, if institutions shift what they report year by year without making that explicit. Only the NIH can rectify this challenge, by specifying a standard unit (number of cages or number of animals) that institutions must report. These “average daily inventory” data are thus not used in this project to develop the total RM usage data.

Another indirect route to establishing US rodent numbers is to extrapolate from species percentages in other jurisdictions that have clearer mandates for transparency and more comprehensive standards on types of use to report^13–18^. Taylor et al*.* combined this approach with extrapolation based on publication rates by species and country, starting with European Union definitions of “animal” and EU categories of reported animal use. They derived a conservative estimate of 17.3 million total animals used in the United States for 2005 and contrary to the increase that Goodman et al*.* reported, a decrease to 14.6 million in 2015^3,4^. Given that just 16 responding US institutions reported some 5.6 million animals to AAALAC in the study reported here, Taylor’s estimate of 14.6 million looks low.

A third approach could be to apply the ratios of RM to other mammals as reported in countries with greater transparency and reporting, without Taylor’s added step of factoring in publication rates. For example, in Germany, RM comprised approximately 92.6% of mammals used in 2018^17,18^. If German data applied to the US system, that would dramatically lower estimated United States RM use from approximately 111 million to approximately 11 million, even lower than Taylor’s estimate. This would mean that 16 responding US institutions of the 30 top NIH-funded used fully half of all RM in the US. The other 14 top-funded sites, plus all less-funded sites, plus industry research and development, plus regulatory safety and efficacy testing plus animals in NIH and other government laboratories would comprise the other half. As the responding institutions used but 5% of the total reported non-RM mammals, it seems implausible they used 50% of the national RM.

Other countries report more clearly than the US on species use overall, but also more granularly, such as Germany’s data on species use by purpose. RM percentages vary with purpose in Germany, so in 2018 “Basic research” used 97.9% RM, “Translational and applied research” used 93.6% RM, “Regulatory use and routine production” used only 76.3% RM whilst “Higher education or training” used 92% RM. There are no US statistics on species use by purpose, nor does it seem that the US uses these same categories. Nonetheless, Germany’s varied use by purpose raises caution that sampling only large academic campuses, mostly of them public institutions, could skew the data presented here.

Readers should bear in mind that German and others’ patterns of use may well differ to those in the US. Scientific norms for choice of animal model and numbers of subjects may be fairly consistent, but regulatory issues can drive differences among countries. Countries set their own requirements for species use in preclinical safety and efficacy testing, and US data are not available to allow a precise comparison, or to explore whether RM are used in greater proportions in US regulatory testing. Nor are there data beyond my own anecdotal experience in academic animal research support that administrators and individual scientists in the US are fully aware that expanded use of AWA-covered animals would open doors to greater government oversight and inspections that they would prefer to avoid. It is presently impossible how much this avoidance of government oversight affects actual species numbers in US research. Thus, international data such as those from Germany do not appear to be directly useful for estimating animal use in the US, but certainly point to patterns of use that are worth exploring.

My choice of sampling method reflects concerns that non-United States data, though far more detailed and transparent, employ different definitions of animal “use,” different categories of “severity” (or likelihood to cause significant animal pain and distress) and may reflect patterns of use different to United States laboratories’ patterns. It must be recognized that my sampling method does not include private industry or research done in government laboratories. Moreover, my sampling method resulted in data from more public institutions, subject to their states’ open records laws, than from private universities and their affiliates. There are insufficient data available from private institutions, whether nonprofit or for-profit, to test for whether there is a systematic difference in species use in these settings. Among the 16 high-funded responding institutions, there was a strong positive correlation between the number of rats and mice and the amount of funding the institution receives (N = 16, r = 0.76), likely reflecting general levels of research activity. There was a weaker positive correlation between the amount of funding the institution receives and percent of total laboratory animals that are rats or mice (r = 0.23), suggesting that this sample of elite institutions may satisfactorily represent academic RM use more broadly.

In the present study, the mean RM percentage was 99.3%, with a range of 97.3% to 99.6%. Applying this range to the 780,070 AWA-covered “animals” yields a range of approximately 21.1 to 195 million RM. As discussed above, very low estimates (21 million) seem implausible given the 5.6 million total RM reported at just 16 institutions. The ranges reported here may reflect true ranges in patterns of animal use, or may reflect imprecision in reporting on animal use, as when institutions estimate their annual animal use based on a conversion factor applied to their standing inventory. Notice how a small difference in RM percentage (99.3 versus 99.6% RM, or reframed as 0.7 versus 0.4% non-RM) changes the estimated totals from 111 to 195 million.

The estimate presented here may over-estimate “use” as that word lacks a clear definition with NIH and AAALAC. The AWA’s C, D and E categories cover the use of animals in experiments, with a separate category B for breeder or other animals not used, or not yet used on the reporting date of the annual report, in experiments. In this study, I did not include USDA Category B animals. Where I have worked, the majority of our animals “used” in category C, D or E would mostly also have been reported that year as Category B animals at the vendor we received them from. The present survey using AAALAC reports was unable to differentiate rodents in service solely as breeders or born but culled for having an unusable genotype, roughly comparable to AWA Category B animals. If “use” is narrowly defined as actual enrollment in an experiment, the estimate here, which is unable to distinguish breeders or other “unused” animals, is too high. More precision would require reporting on breeding programs for transgenic rodents as the EU statistics call for^4^.

It is also possible that 111.5 million is too low an estimate. Evidence here suggests that institutions’ annual reports to AAALAC may undercount RM if they extrapolate from daily cage inventories rather than counting purchased animals plus those born in-house. AAALAC and NIH require some approximate measure of the scope of a program in their efforts to evaluate the adequacy of an institution’s resources devoted to maintaining the animals. Among the responses to this survey, two respondents reported extrapolating daily cage inventory to annual mouse use using a conversion factor of 3 or 3.25. Anecdotally, my colleagues and I found that while one cage on inventory averaged approximately three mice on daily inventory, but closer to ten mice per year, given the turnover of mice occupying the cages. Most respondents were silent on whether they count cages and extrapolate, or count animal births and purchases. Those who extrapolate from their daily cage inventory are likely undercounting total annual RM numbers if they are using a conversion of approximately 3.

AAALAC does not share information from its accredited institutions and USDA does not have RM data, so any attempt to estimate American RM use requires extrapolating from other sources of data. NIH data are available via FOIA requests but are of limited value in that they do not standardize whether to specify if the count is animals versus cages and NIH oversight does not include large pharmaceutical companies and small biotechnology companies if they are not recipients of federal grants. Though it would still only apply to a subset of American laboratories, NIH transparency would be improved if it tallied and published aggregates statistics as the USDA does, especially if it standardizes what numbers institutions must report.

For the present project, I chose to work with American statistics, given how definitions of “animal” may differ, for example in how agricultural and ecological research subjects are counted. Additionally, other countries classify animal use by assessed severity of procedures that animals undergo, unlike the United States system where the reasons for using analgesics and anesthetics, not the actual severity of the project distinguish the various USDA “pain categories”.

In this project, I estimate that over 44 million mice and rats are in painful or distressful experiments. This estimate relies on the only available United States pain classification, that of AWA-covered animals, and depends on accurate self-reports from institutions Given the challenges of identifying and treating rodent pain, and that many animals in Category D may be under-treated for pain, there is limited value in distinguishing categories D and E^19^. In fact, somewhat counter-intuitively, an animal who undergoes a day of food deprivation is in Category E whereas an animal undergoing multiple major survival surgeries, as long as they are anesthetized, is in Category D. The accuracy of this estimate depends on the premise that the relative proportions of C, D and E animals are similar in RM as they are in AWA-covered animals; this may be incorrect in either direction. On the one hand, RM are extensively used in studies with significant unalleviated pain and distress such as psychiatric distress models, advanced cancer, sepsis, infectious disease, and pain biology, so numbers on painful studies may be higher than reported here. On the other hand, by including breeders, culled animals, and euthanized tissue donors who do not undergo painful experiments in the 111.5 million may mean proportionately fewer of the RM are in the higher pain categories.

There is broad interest in knowing the numbers of animals in research. Congress first mandated reports in 1970, long before it stipulated that RM are not “animals” in research ^14^. Research defense organizations as well as animal protection organizations post statistics on animal numbers, reflecting their belief of what the public wants to know, but until now have not had data on which to base their statistics^7,20–24^.

The present estimate of 111.5 million rodents is higher than others available; the true number could be higher or lower. This single number at one time-point has limited value, but establishes a benchmark for monitoring trends, assuming future reviews use the same methodology with the same assumptions. It appears likely that year by year, use of AWA-covered species is gradually decreasing^10^ while RM numbers are likely increasing, but as this is the first evidence-based estimate of United States mouse and rat use this study cannot demonstrate a trend, as valuable as that could be for future efforts to analyze increases and decreases in animal use.

The AWA is a powerful tool for laboratory animal welfare, with its system of inspections, whistleblower investigations, coverage of animals in private as well as public institutions and its transparent reporting of animal use statistics. Many have argued that the large numbers of RM, especially in the most painful experiments, warrant their inclusion as AWA animals along with the primates, dogs, hamsters and others currently counted as animals^1,22–27^. Others, including the USDA itself, may see these numbers as evidence that this evolution would be beyond the USDA’s resources, adding not just more animals per inspected facility, but also covering whole RM-only facilities previously not regulated by the AWA. Some may take comfort that research seems to be shifting more and more away from larger animals or so-called “higher-order species” toward mice and rats. Others may be discomfited if that equates to overall increases of sentient animals in laboratories.

Defining RM as “animals” would require congressional amendment of the AWA. NIH initiatives to standardize reporting, differentiating RM on painful procedures from others, and publishing aggregate statistics would not.

Rodents’ capacity to experience significant pain and distress in experiments is no longer contested. With over 100 million of these sentient animals born per year for American science, it is time to revisit the adequacy of their welfare protections.

## Data Availability

All data is available within the manuscript.
